# Methods and Tools in RNA Biology

**DOI:** 10.3390/ncrna9040046

**Published:** 2023-08-10

**Authors:** Mirolyuba Ilieva, Shizuka Uchida

**Affiliations:** Center for RNA Medicine, Department of Clinical Medicine, Aalborg University, DK-2450 Copenhagen SV, Denmark

Breakthroughs in innovative techniques and instruments have driven the exploration of non-coding RNAs (ncRNAs), including microRNAs (miRNAs) and long non-coding RNAs (lncRNAs). Whenever the data collected is at a transcriptional-wide scale, it becomes crucial to utilize computational power to identify ncRNAs. Moreover, the information extracted from this experimental data needs to be annotated to associate it with existing knowledge and aid in the further detection of new ncRNAs. After transforming these data into meaningful information, further data processing is essential to share this acquired knowledge with a broader community, thereby enabling more scientific discoveries. In this Special Issue, experimental techniques, and tools for ncRNAs, as well as computational approaches to process, annotate, classify, and distribute this information to shed more light on the role of ncRNAs, are shared.

MicroRNAs (miRNAs) are small, single-stranded RNA molecules, approximately 22 nucleotides long. They play critical roles in RNA silencing and the regulation of gene expression following transcription. Given that a single miRNA can bind several hundred messenger RNAs (mRNAs), they impact numerous signaling pathways. Moreover, disruptions in miRNA regulation have been associated with the development of diverse diseases, underlining their significance in maintaining the normal physiological functions of an organism. As such, there are various bioinformatic tools available to analyze miRNA sequences, perform target prediction/validation, associate miRNAs to diseases, perform pathway analysis, and discover genetic variants within miRNAs. Since there are many useful tools available, it is a daunting task to search and investigate each tool. To address this point, Buitrago et al. nicely summarize the currently available online tools for miRNAs [1].

As we accumulate biological experimental data, more information about miRNAs can be obtained. Having more data does not necessarily mean we know more about miRNAs, unless these data are cross-validated with other existing data. As the main objective of bioinformatic approaches is to combine different types of data to discover something new, it is no exception that new algorithms and methods are being continuously developed for miRNA research. From this perspective, Sayed and Park introduced a R Shiny application, miRinGO (https://github.com/Fadeel/miRinGO, accessed on 4 August 2023), to detect biological processes indirectly targeted by miRNAs transcriptionally through transcription factors, even if there is no physical interaction between miRNA and the regulated genes [2].

Dysregulation of miRNAs has been linked to various diseases. Thus, there is an intense interest in studying the association between miRNAs and diseases. As there are over 2000 miRNAs in humans, it is often difficult to pinpoint which miRNA is important for a particular disease. To solve this problem, Wang and McGeachie introduced a novel computational method, DisiMiR (https://github.com/Wanff/DisiMiR, accessed on 4 August 2023), which predicts pathogenic miRNAs by inferring biological characteristics of pathogenicity, including network influence and evolutionary conservation [3].

Besides miRNAs, there is a growing interest in studying much longer ncRNAs, namely lncRNAs, whose lengths are longer than 200 nucleotides. Compared to protein-coding genes, the number of lncRNAs is several times higher as they are expressed in a certain cell type but not the others. Also, they are often dysregulated under stressed conditions and diseases, suggesting their potential involvement in the pathogeneses of various diseases. Roughly half of lncRNAs own poly A tails, which enables them to be discovered from RNA sequencing (RNA-seq) data as this technique captures gene expression at a particular time point in an unbiased manner. If the most abundant transcripts in a cell (i.e., ribosomal RNAs (rRNAs)) are depleted in prior to sequencing library construction, lncRNAs without poly A tails can also be detected. As the price for performing RNA-seq experiment has dropped significantly in recent years, RNA-seq assay has been used as a primary screening tool to analyze the transcriptome of various cell types and tissues, developmental stages, and diseases, including patients’ samples. Unfortunately, most of these RNA-seq data are analyzed only for protein-coding genes but not for lncRNAs. However, many of these RNA-seq data are available online in public domains (e.g., Gene Expression Omnibus (GEO)) as scientific community pushes for the sharing of generated data after publication of the original studies. Thus, such RNA-seq data are gold mines for discovering lncRNAs that are dysregulated in a particular disease with well characterized clinical data that have been peer-reviewed by editors and reviewers of prominent journals. To utilize such RNA-seq data from patients suffering from diseases, Ilieva et al. conducted a secondary analysis of the published RNA-seq data from patients suffering from nonalcoholic fatty liver disease (NAFLD) to discover dysregulated lncRNAs [4]. To disseminate the newly acquired knowledge about lncRNAs in NAFLD, a knowledge database, LiverDB (https://rnamedicine.shinyapps.io/liverdb/, accessed on 4 August 2023), was introduced.

Significant progress in cancer treatment has led to the emergence of cardio-oncology, which aims to minimize cardiovascular risks while maximizing cancer therapy effectiveness. However, treatment-induced cardiotoxicity remains a prominent health risk. Among these cancer drugs, doxorubicin is notoriously known to cause cardiotoxicity, which may ultimately lead to death or cardiac transplantation. Thus, numerous approaches, including RNA-seq, have been employed in various cell types and tissues to uncover the side effects of doxorubicin treatment. To further understand the impact of doxorubicin, Distefano et al. conducted a systematic analysis of published RNA-seq data to discover doxorubicin-induced lncRNA genes [5]. By performing loss-of-function experiments in cardiac fibroblasts treated with doxorubicin, it was found that the lncRNA *MAP3K4-AS1* might play a protective role against doxorubicin toxicity. To further disseminate the analyzed data, Distefano et al. built a web database DoxoDB (https://rebeccadistefano.shinyapps.io/DoxoDB/, accessed on 4 August 2023).

Although discovering lncRNAs from the existing RNA-seq data is important, it is much more challenging to uncover the functional roles of lncRNAs. Slowly but surely, more lncRNAs have been functionally and mechanistically studied, including their functions achieved via binding to DNA, RNA, and proteins to regulate epigenetic, genomic, transcriptomic, epitrancriptomic, and proteomic events. Within the nucleus, the engagement of lncRNAs with DNA and the resulting creation of atypical nucleic acid configurations appear to be especially pertinent. Apart from interactions between single-stranded RNA (ssRNA) and single-stranded DNA (ssDNA), like R-loops, ssRNA can also associate with double-stranded DNA (dsDNA) to constitute DNA:DNA:RNA triplex structures. A current challenge in studying DNA:DNA:RNA triplexes is the identification of the precise RNA component interacting with specific regions of the dsDNA. To this end, computational methods are useful to predict such sequences, which Warwick et al. nicely summarized [6].

Taken together, this Special Issue highlights the importance of computational methods and tools for studying miRNAs and lncRNAs. Besides introducing new algorithms and bioinformatic tools, including databases, this Special Issue includes review articles that provide tables of useful bioinformatic tools to further advance the field of ncRNAs, which researchers could use to screen a set of disease-relevant lncRNAs in silico and to design further biological experiments based on computational predictions.
